# Stockholm preterm interaction-based intervention (SPIBI) - study protocol for an RCT of a 12-month parallel-group post-discharge program for extremely preterm infants and their parents

**DOI:** 10.1186/s12887-020-1934-4

**Published:** 2020-02-01

**Authors:** Erika Baraldi, Mara Westling Allodi, Kristina Löwing, Ann-Charlotte Smedler, Björn Westrup, Ulrika Ådén

**Affiliations:** 1 Department of Special Education, Specialpedagogiska institutionen Stockholms universitet, Stockholm University, Frescati Hagväg 10, 106 91 Stockholm, Sweden; 2 Department of Women’s and Children’s Health, Institutionen för kvinnors och barns hälsa, Karolinska Institutet, Karolinska Institutet, 171 77 Stockholm, Sweden; 3 Functional Area Occupational Therapy & Physiotherapy, Allied Health Professionals Function, Karolinska University Hospital, 171 76 Stockholm, Sweden; 4 Department of Psychology, Psykologiska institutionen Stockholms universitet, Stockholm University, Frescati Hagväg 8, 106 91 Stockholm, Sweden; 5 Neonatology unit, Karolinska University Hospital, 171 76 Stockholm, Sweden

**Keywords:** Child cognitive development, Child motor development, Early intervention, Emotional availability, Extreme prematurity, Parent-child interaction, Parental mental health, Self-regulation

## Abstract

**Background:**

Improved neonatal care has resulted in increased survival rates among infants born after only 22 gestational weeks, but extremely preterm children still have an increased risk of neurodevelopmental delays, learning disabilities and reduced cognitive capacity, particularly executive function deficits. Parent-child interaction and parental mental health are associated with infant development, regardless of preterm birth. There is a need for further early interventions directed towards extremely preterm (EPT) children as well as their parents. The purpose of this paper is to describe the Stockholm Preterm Interaction-Based Intervention (SPIBI), the arrangements of the SPIBI trial and the chosen outcome measurements.

**Methods:**

The SPIBI is a randomized clinical trial that includes EPT infants and their parents upon discharge from four neonatal units in Stockholm, Sweden. Inclusion criteria are EPT infants soon to be discharged from a neonatal intensive care unit (NICU), with parents speaking Swedish or English. Both groups receive three initial visits at the neonatal unit before discharge during the recruitment process, with a strengths-based and development-supportive approach. The intervention group receives ten home visits and two telephone calls during the first year from a trained interventionist from a multi-professional team. The SPIBI intervention is a strengths-based early intervention programme focusing on parental sensitivity to infant cues, enhancing positive parent-child interaction, improving self-regulating skills and supporting the infant’s next small developmental step through a scaffolding process and parent-infant co-regulation. The control group receives standard follow-up and care plus extended assessment. The outcomes of interest are parent-child interaction, child development, parental mental health and preschool teacher evaluation of child participation, with assessments at 3, 12, 24 and 36 months corrected age (CA). The primary outcome is emotional availability at 12 months CA.

**Discussion:**

If the SPIBI shows positive results, it could be considered for clinical implementation for child-support, ethical and health-economic purposes. Regardless of the outcome, the trial will provide valuable information about extremely preterm children and their parents during infancy and toddlerhood after regional hospital care in Sweden.

**Trial registration:**

The study was registered in ClinicalTrials.gov in October 2018 (NCT03714633).

## Background

Being born extremely preterm, i.e., born before 28 gestation weeks, is a potentially life-threatening circumstance affecting the child [1–6], the parents [7–10] and the interactions among family members [11–13]. Swedish health care delivers high quality services to all citizens regardless of family income and offers active and advanced neonatal intensive care, saving 90% of children born extremely preterm [14]. In Sweden, 0.3% of all children are born extremely preterm (EPT), and the Swedish Federation for Preterm Infants (SPF) stresses that surviving EPT children constitute a new group of patients in need of support beyond the intensive care period [15]. Further highlighting the urgency for additional supportive care to families with EPT-born infants, Sweden’s frontline neonatal care [16] has resulted in a new EPT population of surviving children born as young as 22 + 0 to 23 + 6 gestational weeks, and the long-term outcomes for this novel population are not yet known.

Swedish data show that approximately 2/3 of the EPT children have no or mild impairment, while 1/3 have moderate to severe neurodevelopmental impairments when entering primary school [4], with signs thereof already in preschool [5]. EPT children are a defined high-risk population, and the occurrence of cognitive impairment [1] increases the earlier in pregnancy the child was born [17]. Working memory [18] and executive functions [3, 19, 20] seem to be particularly vulnerable in extremely preterm children, and Attention Deficit Hyperactivity Disorder (ADHD) is twice as common among EPT children compared to term peers [21]. Executive function (EF) deficits are of particular developmental interest, since self-regulation is associated with EF [22], and EPT children tend to display early self-regulatory difficulties [23]. Another neuropsychiatric disorder overrepresented among EPT children is autism spectrum disorder (ASD), which is diagnosed in 17% of EPT children. In some studies, up to 29% of EPT children screen positive according to ASD observation protocols [24–26]. Moreover, several skills that are important for school success, such as mathematical [27] and linguistic abilities [28, 29], are negatively affected by extreme prematurity; preterm-born students in general do not perform at the same level as their term classmates in school [30–32]. Additionally, EPT children have an increased risk of mental illness [33, 34] and of being bullied throughout school [35]. Given the described outcomes of extreme prematurity, there is a clear need for interventions and treatments that may positively influence the long-term development of EPT children.

Prematurity affects not only the individual child but also the family as a whole. Giving birth unexpectedly early, missing part of the pregnancy, the fear of losing the child, long-term stays at the neonatal intensive care unit and marital challenges are amongst some of the commonly referred strains with which parents of preterm infants must cope. From a longer-term perspective, after discharge, new challenges often occur: the question of how to support the child optimally upon coming home; how to interpret the often-more-diffuse behavioural communication of the EPT infant compared to that of infants born at term age; how to patiently wait for, identify and support the next developmental step of the child; and how to feel competent as a parent at home. Parental mental health may be negatively affected by a child’s preterm birth [8, 36], and poor parental mental health is associated with less favourable social, behavioural and functional development of preschool-aged EPT children [37, 38]. Hence, adequate discharge planning and transition programmes for the child and family leaving the Neonatal Intensive Care Unit (NICU) is an area in need of further development [39, 40] to benefit not only the child but also the parents.

An important concern with regard to the post-discharge programme for the EPT population is the most appropriate content of such an intervention. International efforts have been made to summarize effective qualities of interventions for the general population [41], and in 2015, a Cochrane report was published that charted the post-discharge programmes for preterm-born children [42], concluding that the post-intervention programme should target both motor and cognitive outcomes and that programmes focusing on providing an optimal environment for learning have accumulated more evidence. A later meta-analysis indicated that interventions given both in the home and in the hospital/preschool show the most promising results [43], suggesting that there should be multiple locations. Similar to term children and their parents, EPT children are dependent upon their caretakers throughout their upbringing; therefore, interventions targeting both infants and parents as well as their relationship might be more effective than interventions with unidimensional targets. It is hardly surprising that an EPT birth influences the infant-parent relationship unfavourably [11, 44], and parental behaviour should therefore also be targeted. Since parental responsivity seems to be the parental style that influences preterm children’s cognitive development the most, and since parental responsivity and warmth seem to affect preterm children’s behaviour favourably [45], these should be critical components of any post-discharge intervention aimed at this group. Moreover, the finding that parental rejection affects indicators of preterm children’s behaviour negatively [45] supports the idea of a strengths-based approach, focusing on children’s abilities more than their difficulties. In addition, attention should be given to the EPT population’s challenges concerning executive functions. Since self-regulation is associated with executive development, helping the preterm child to self-regulate should be an essential part of a post-discharge programme. Executive function is, in turn, crucial for the maturation of social skills [46] and academic achievement [47] of all children.

**Fig. 1:**
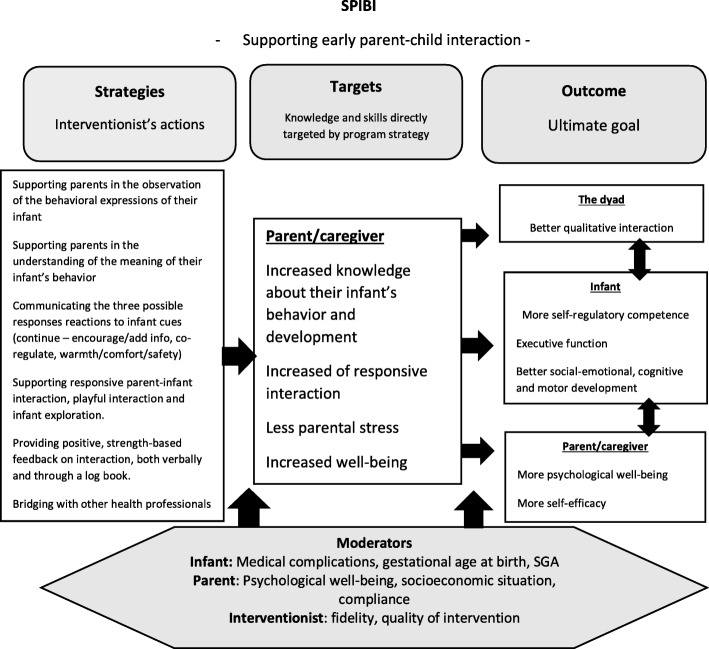
SPIBI theory of change

Internationally, different post-discharge programmes have displayed different approaches of the abovementioned ideas of intervention content, for example, the Infant Behavioral Assessment and Intervention Program (IBAIP), the ToP programme [48–51], the modified Mother Infant Transaction Program (MITP) [52, 53], the Infant Health and Development Program (IHDP) [54] and a Taiwanese home-based intervention programme [55], among others. Different programmes offer intervention visits at different times in the discharge process and with different content. The MITP builds on sensitizing parents to baby cues and at the same time introducing them to stimulating activities for their infants. Most of the visits are scheduled during the last week of their hospital stay, with two additional home visits during the first quarter of a year at home [52]; hence, the intervention focus is rather early in the discharge process. The IBAIP is described as a strengths-based intervention, building on both infant and parental qualities and enhancing self-regulatory and co-regulatory behaviour [48], and in the Dutch trial of the IBAIP, it consists of 6–8 home visits from an infant physiotherapist before 6 months CA. The Dutch research team later developed the ToP intervention, which is now a part of standard care for very preterm children and consists of 12 home visits during the first year at home; hence, the focus of the intervention is slightly later that in the case of the MITP. Other programmes, such as the IHDP, are more extensive and last until 36 months CA, including home visits, an educational child care programme and a bimonthly parental group during the last 2 years of the intervention. The home visits introduced both age-appropriate games for development and family support of parent-identified problems. Post-discharge interventions are not exclusively tested in Western societies; for example, a Taiwanese research group conducted up to 13 visits in the clinic or home environment during the child’s first year to teach child developmental skills, provide instruction on health-related topics and feeding and massage procedures, support parents and enhance parent-child interaction, which showed positive results on infants’ emotion regulation and stress responses in toddlerhood [55]. Many of the cornerstones of these programmes are also included in the SPIBI, which can be seen in the theory of change of the SPIBI (Fig. 1).

Stockholm County has four NICUs, where all professionals work to individually adapt intensive care in accordance with the Newborn Individualized Developmental Care and Assessment Program (NIDCAP) [56]. However, the NIDCAP, which supports parents’ ability to read and adequately respond to baby cues, unfortunately ends at discharge. To contribute to a cohesive chain of care, the SPIBI builds on the same principles as the NIDCAP, e.g., relating to the synactive theory [57].

We designed a randomized clinical trial to evaluate the effect of an interaction-based programme for EPT infants and their caretakers, beginning in the discharge period and lasting until the child is 12 months CA. The aim of the SPIBI is to give this fragile population of children a better start in life by improving the quality of parent-child interaction and by supporting the parents. The programme is in line with the SFP’s call for enhanced post-discharge support. The intervention aims to implement treatments for the new group of EPT survivors that require specific competences and responsiveness acquired through building on existing knowledge of international post-discharge interventions. The intervention is designed to have the following qualities: to match the unique Swedish context, with infants being saved from being born in earlier gestation weeks; to include a rather extensive follow-up programme; and to provide outcome data from an EPT population, with free healthcare services and 480 days of parental leave per child.

## Methods

The study was designed in accordance with the SPIRIT 2013 statement.

### Aims

This study aims to examine the effects of Stockholm Preterm Interaction-Based Intervention (SPIBI) in three overall domains: parent-child interaction, child development and parental mental health. The aim of the present paper is to give the rationale, content and trial design of the SPIBI.

### Hypotheses

The primary hypothesis of the SPIBI is that the quality of the parent-child interaction will improve, and more specifically, that the emotional availability of both the child and parent will be higher in the intervention dyads than in the control dyads post-intervention.

The secondary hypotheses concern the children and the parents, respectively. The secondary hypotheses concerning the children are that the children in the intervention group will have enhanced development compared to the control children during the intervention, with an enduring effect concerning their general development, executive function, motor development, neurological development and autistic symptoms. Additional secondary hypotheses are that when preschool teachers are asked about their view of their extremely preterm pupil, they will describe the children in the intervention group as more participatory and playful than the control children are.

The secondary hypotheses concerning the parents are that compared to parents of children in the control condition, parents of the children in the intervention condition will be less depressed, less anxious and more resilient to stress, as well as describe themselves as having higher parental self-efficacy.

All hypothesises are formulated as objectives with specific outcomes attached to them as well as period of activities in Table 1.

**Table 1:** Project framework: specific objectives, outcomes and period of activities

Specific Objectives	Outcomes	Period of activities
Objective 1:		
To improve the quality of the interaction between parents and their extreme preterm children	All participants will be filmed during a free play situation during a 20 min interaction. The video films will be analyzed using the Emotional Availability Scales (EAS).	Parent-child interactions will be video filmed at 12 months corrected age (at the end of the intervention) and again at 2 years corrected age (1 year post-intervention).
Objective 2:		
To improve child development within several areas	Child’s general development (BSID-III, ASQ, SDQ), Child executive function (BRIEF-P), Child’s motor development (AIMS, PDMS, GMA), Child’s neurological development (HINE, HANE), child’s autistic symptoms (M-CHAT), child’s temperament (IBQ-R), preschool teachers view of their extreme preterm pupil (CEQ, “Ert barn vårt samspel”, playtime/social time impression scale, ICF-CY and semi-structured interview with the preschool teachers).	Motor skills assessment will begin at 3 months corrected age at the neonatal follow-up unit. Other child outcomes will be measured at 1, 2 and 3 years corrected age, in accordance with the age range the assessment and questionnaires are applicable for. Preschool teachers’ view of their extreme preterm pupils will be collected at 2 or 3 years corrected age, depending on preschool introduction for that specific child.
Objective 3:		
To improve parental mental health of parents to extreme preterm infants post-discharge	Parental mental health (STAI & HADS) and views of parenthood (PSE & RES).	Parental questionnaires will be collected at baseline and when their child has reached an age of 1, 2 and 3 years corrected age.
Objective 4:		
Collecting parental views of the first year at home after NICU-discharge with an EPT infant; both intervention and control group.	Semi-structured interviews with a focus of the first year at home, strengths and challenges. CSQ from intervention group.	Post intervention at 1 year corrected age.

### Content of the programme given to the intervention group (IG)

The SPIBI is a manualized, strengths-based home-visit programme focusing on parent-child interaction, skill in reading children’s cues and the provision of optimal support for children’s next small developmental step (see Table 2), including the elements of verbal praise and special play [41]. The basic idea is to reduce the amount of time children spend in a stressed state, which may be toxic to the infant brain, and to enhance developmentally appropriate parent-child interaction to achieve mutual enjoyment. Increased parental self-efficacy is considered to be a common mediator of family-centred practices in early childhood intervention [58], and the parent’s behaviour is a central target. The brief description of the visits below is a condensed version of the 50-page Swedish SPIBI manual specially developed for this trial.

**Table 2:** Intervention content in brief

Cornerstones of SPIBI	
1.	Strength-based support of parent-child interaction
2.	Sensitizing parents to infant cues
3.	Giving optimal support for the child’s next developmental step through scaffolding
4.	Enhancing self-regulating and co-regulation

The purpose of the first visit at the neonatal unit or hospital ward where the child is still being treated is to give the parent (s) a chance to get to know the interventionist and show her the environment where the infant has spent his/her first 3–5 months of life. The interventionist initially explains the scope of the home visits and briefly describes the intervention to the parent (s), with a clear definition of what distinguishes the intervention from regular follow-up care in Stockholm for EPT children. The logbook that will be used during the home visits is presented to the parents; this logbook emphasizes playful interaction, striving for reciprocal amusement and intersubjectivity [59–62] and providing developmental support in the child’s proximal zone of development [63]. All formalities are carefully written down, i.e., contact information, the time of the next home visit and the manner in which the home visits will be recorded in the logbook for the parents as well as in the medical records for the healthcare professionals.

Home visits 1–3 and two telephone calls are provided before the child is 3 months corrected age. The focus of these home visits is to observe the child and parent at home, validate the child’s strengths and competences and enhance parent-child interaction, building on strengths. The child’s strengths and interests will be summarized in the parents’ logbook. All feedback to parents, presented orally as well as in written form, is given in a positive and non-judgemental way. During the three initial home visits, the focus is to confirm the child’s competencies, pointing to the child’s capacity to self-regulate, the child’s individual temperament, and early communication. Infant behaviour is categorized into one of three levels of stability, all with different optimal parental responses: red-labelled stress behaviour, in which the proper parental response is to offer calmness, comfort and safety; yellow-labelled concentration/coping and calming behaviour, in which the adjusted parental response would be to respect the infant’s need for a break or co-regulate him/her; or green-labelled approaching behaviour, in which the child should be offered new information or stimuli to develop further. The interventionist pays extra attention to the child’s arousal level, naming the child’s current tiredness or alertness with the correct term according to Als’ original definitions [57], and upon specific parental inquiry, generally informing parents about preterm children’s tendency to be fussy and discontent in an intermediate stage of sleep and awareness, before more distinct and stable levels of arousal are developed. All visits are scheduled according to the manual in chronologic time +/− 3 weeks to individualize support to the family needs and wishes.

During home visits 4–8, the interventionist, step-by-step and always with the utmost respect to the child’s level of development, will help the parent find suitable objects/toys at home for the infant to examine with the mouth, hands and body, as well as confirm the infant’s abilities and give suggestions for the stimulation of further development in the infant’s interaction with the parent. The logbook will now also contain suggestions for supporting the next developmental step, which will be formulated by the interventionist together with the parent.

**Fig. 2:**
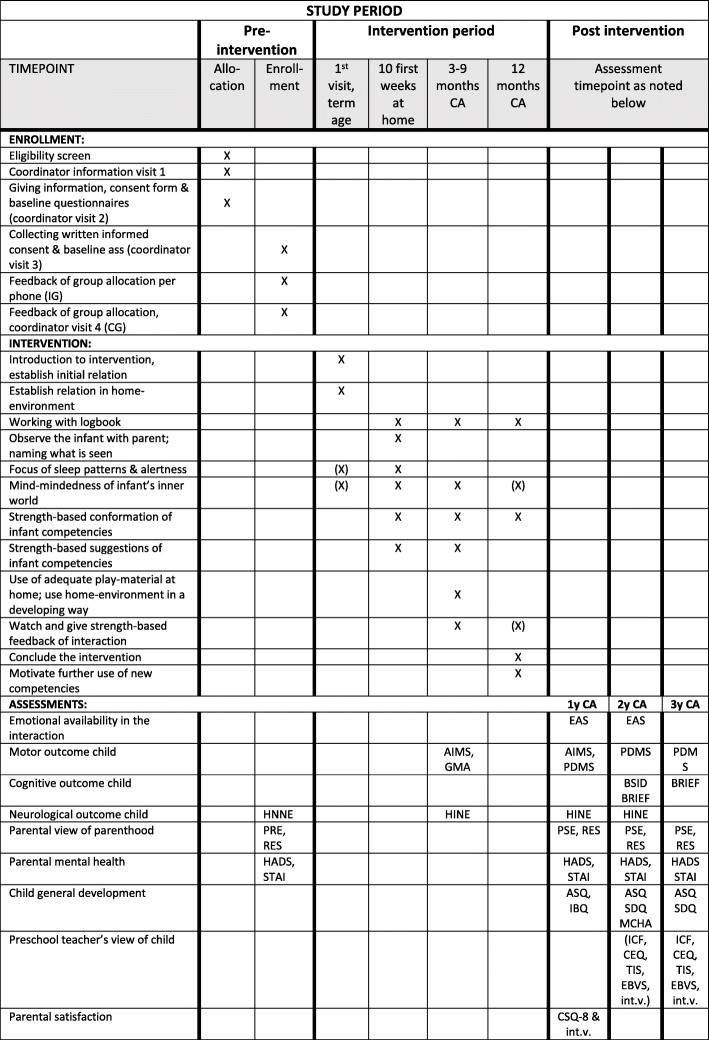
Study enrolment, intervention and assessment. (X) indicates that it is optional to include at this time-point

When the child is 12 months corrected age, the interventionist makes the 9th and final home visit, emphasizing the child’s progress during the past year, looking through the logbook with the parent (s), summarizing the past year and talking about the next developmental step for the future. The logbooks are not used for research, but only for individual parental development. Since the intervention is interactive and relation-based, there is an ethical as well as a pragmatic need for a clear finishing phase to encourage parent for future use of the intervention strategies, for which the logbook is a useful tool. Further programme features are provided in Fig. 2.

### Control group (CG)

The intention of the control condition is that they will receive treatment as usual (TAU). The TAU in Sweden for EPT-born children consists of home-care nursing visits as long as the infant is tube-fed or in need of extra oxygen supply. The recommended basic follow-up for high-risk children in Sweden includes a standardised doctor’s examination at full term, hearing screening and ophthalmologist assessment. At 3 months CA, a physiotherapist and paediatrician assess the infant’s early motor development and neurological progress. Additional follow up visits are common during the first year upon clinical indication. At 1 year CA, the child re-visits the paediatrician and physiotherapist for further motor and neurologic assessment. Throughout the care chain, the paediatrician may refer the patient to the neurologist, pulmonologist, gastroenterologist or child habilitation centre if indicated. At 2 and 5.5 years of age, the child is assessed by a psychologist, paediatrician and physiotherapist. The psychologist assesses the child’s cognitive level and screens for communicative and behavioral problems. The neonatal follow-up team collaborates closely with a speech and language therapist, an occupational therapist and a dietician, all of whom may join the team assessment if necessary. Concerning the SPIBI control group, the recruitment process implies approximately three coordinator visits, four baseline questionnaires of the parents and one extra child physiotherapy assessment. All study participants, controls and intervention children will receive an extended follow-up programme, with additional assessment and questionnaires at term age, 3 months corrected age, 12 months corrected age, 24 months corrected age and 36 months corrected age. In addition, all EPT children in Stockholm are offered a standard follow-up programme and will be referred to specialized care when needed.

### Procedures to implement the intervention

The six interventionists (see Table 3) have professionally diverse backgrounds with several years of neonatal unit experience and have been carefully selected by the research team. The SPIBI training was conducted 1 day per week from October 2017 to October 2018, consisting of theoretical lectures, practical intervention-focused days, and at least six home visits to four different preterm-born children for every interventionist, including subsequent supervision. Each home visit was video-recorded and was then analysed and discussed during supervision. The theoretical lectures were given by Swedish and international researchers and clinicians specializing in fine and gross motor skills development, the cognitive development of preterm children, brain development, the NIDCAP, attachment, parental perspectives, early interventions, special education, early intervention for children with autism, parental mental health, play and interaction, speech and communication development, eating development, strengths-based support and long-term consequences of prematurity. The practical and intervention-focused part of the training were carried out by two infant physiotherapists with clinical and research experience with preterm children through the development of the Dutch post-discharge early intervention programme ToP [48, 49, 51, 64], Karen Koldewijn and Marie-Jeanne Wolf from Amsterdam Academic Medical Centre in Holland.

**Table 3:** Multidisciplinary team of SPIBI

Member of the team	Role in the team
Professor of Special education	PI of research team, main supervisor of PhD student
Neonatologist, professor of neonatology	Research team main medical researcher, facilitator of the project at the NICUs
Pediatric physiotherapist, PhD in physiotherapy	Research team member, supervisor of interventionists concerning motor development and facilitation
Professor emerita of psychology	Research team member, senior advisor of psychology research in neonatal research environment
Psychologist, PhD student	Research team member, coordinator of recruitment, supervisor of interventionists concerning psychological development and attachment
Neonatologist, PhD in neonatology, NIDCAP-trained	Research team member, senior advisor of early intervention in NICU setting
Neonatal nurse, NIDCAP certified	Interventionist, SPIBI-training graduate
Neonatal nurse, physiotherapist, NIDCAP trained	Interventionist, SPIBI-training graduate
Neonatal home-care children nurse	Interventionist, SPIBI-training graduate
Music therapist	Interventionist, SPIBI-training graduate
Psychologist	Interventionist, SPIBI-training graduate
Physiotherapist	Interventionist, SPIBI-training graduate

### Trial design

The SPIBI trial is a two-arm randomized trial with four recruiting sites in Stockholm. The intervention group (IG) receives 10 visits and two telephone calls from a special trained interventionist (see Table 3). The focus of the intervention is providing strengths-based support of the parent-child interaction, sensitizing parents to infant cues, helping the parent to give optimal developmental support to the infant and enhancing the infant’s self-regulating skills. All extremely preterm children in Stockholm are routinely offered an extensive follow-up test programme, and SPIBI participants are subject to additional assessments at 3 months, 12 months, and 24 months corrected age. Additionally, the children’s preschool teachers will be interviewed when they reach 36 months corrected age. Control participants will have an additional meeting with the project coordinator when they are informed that they have been allocated to the control group, in which information will be provided about the discharge process, their child’s behavioural cues and the importance of parent-child interaction at home. The trial began on 1 September 2018, and recruitment is anticipated to end on 31 August 2020 or at a later date when the target 130 is reached. The intervention will continue for 1 year after the last participant has been included.

### Study setting

The study setting will be conducted mainly in the participants’ home environment, except for the first visit, which is intended to occur in the hospital setting before discharge, if applicable.

### Sample size and statistical power

The hospitals in Stockholm treat more than 100 extremely preterm infants every year, but several of them are not residents of Stockholm County, which is a prerequisite for study inclusion. The study team is prepared to recruit 130 participants, 50% of which will be randomized to the SPIBI intervention. The sample size is based on feasibility, and the assumption is that the effect size of the intervention on the primary outcome measure Emotional Availability Scales (EAS) will be moderate, i.e., Cohen’s d = 0.5. This is largely in line with the results of Flierman et al. [50] for the sensitivity scale of the EAS in the previously mentioned Dutch trial. Hence, we aim to recruit 130 participants, which gives us a power of 0.8 given a normal distribution and an alpha value of 0.05.

### Participant inclusion and exclusion criteria

Parents of all EPT children residing in Stockholm County who meet the inclusion criteria will be approached by the end of their child’s hospital stay. Inclusion criteria are that the child was born before 28 gestational weeks (GW), is currently in stable medical condition, and is therefore close to hospital discharge from one of the four neonatal units belonging to the Stockholm Region: Karolinska Hospital Huddinge, Karolinska Hospital Solna, Karolinska Neonatal Unit Danderyd and Sachsska Childhood and Youth Hospital. Exclusion criteria are parents who are not able to communicate in Swedish or English, patients not residing in Stockholm County and acute surgery patients who will spend a substantial amount of time in hospitals far from Stockholm.

### Recruitment and randomization

#### Recruitment

The PhD student working with the project spends 8–20 h per week as a project coordinator (see Table 3) visiting the four neonatal units and two medical child wards to identify families eligible for recruitment. The standard procedure for recruitment includes three visits. The first visit takes place during GW 32–36 and aims to provide initial information about the discharge process in general, including the neonatal follow-up programme and the SPIBI project in particular. This initial visit is only implemented if the nurse or neonatologist in charge of the child considers the patient to be medically stable. Two days to three weeks later, a second visit will take place, during which parental questions are answered and the intervention programme as well as the conditions for participating in an RCT are explained in detail. It is stressed that research participation is voluntary and that the family may withdraw from the project at any time with no further consequences. Participating parents are given a three-page information sheet and a consent form to sign, as well as four baseline assessment questionnaires. During the third visit, informed consent and baseline questionnaires are collected, and information about the project is repeated if necessary. The participant is randomized, and if assigned to the control group, a fourth visit is needed to provide information about this circumstance, as well as the fact that the child and parents are now a part of an extended follow-up starting at 3 months corrected age, and at 1 year corrected age, the PhD student will see the family again for assessment and a follow-up interview. If the participant is assigned to the intervention group, the assigned interventionist will visit the family as soon as possible.

#### Randomization

The Professor Emerita of Psychology (see Table 3), who is not involved in recruitment, has block randomized 130 participants using an Internet-based random generator (http://www.randomization.com). The instructions given to the random generator are separately stored and the procedure will be cross-checked when all participants have been randomized. All families agreeing to participate in the SPIBI are assigned a serial number 1–130 in chronological order from the date on which they signed the informed consent, and this information is stored in a safe locker separate from the baseline questionnaires.

**Fig. 3:**
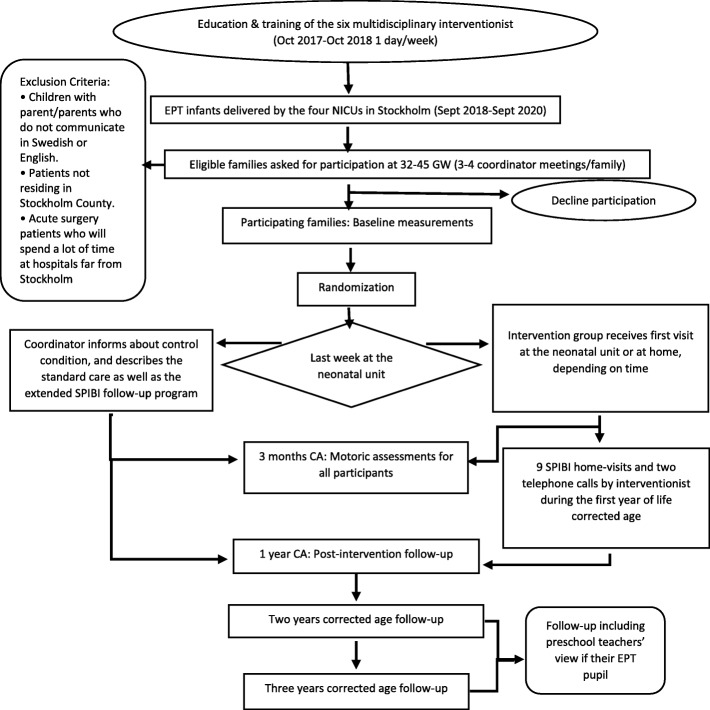
Study flow chart of SPIBI

An overview of the flow chart of the study, including the recruitment process may be found in Fig. 3.

### Evaluation methods

#### Fidelity check

Following each home visit, the interventionist completed a fidelity check of seven questions and one self-evaluation on a scale from 1 to 10 of the interventionist’s faithfulness to the manual during the home visit.

#### Outcome evaluation

The contents of the outcome measures are threefold: parent-child interaction concerning emotional availability, the child’s development and parental mental health.

#### Parental satisfaction

Since the Swedish Preterm Federation has expressed a need for a post-discharge programme and thus stimulated the development of the SPIBI, the views of the participating parents of the benefits and weaknesses of the intervention is of particular importance. All parents will be asked to participate in a semi-structured interview concerning parental satisfaction after the intervention, in addition to rating the intervention with the Client Satisfaction Questionnaire (CSQ-8).

### Outcome measurements

Since the SPIBI is a multi-professional intervention, the outcome is measured across several domains: outcomes concerning emotional availability in the dyadic relation, outcomes measuring child development and outcomes related to parental mental health and parenthood.

The *primary outcome* is the Emotional Availability Scales (EAS) [65], which will be used primarily at 12 months corrected age but then again at 24 months corrected age. The primary coder is blind to group allocation, whereas the second coder, who is used for interrater reliability checks in 20% of the cases, is not. The scale has four parental dimensions (sensitivity, structure, non-intrusiveness, and non-hostility) and two child dimensions (child responsiveness and child involvement). Each subscale has a maximum score of 29 and a direct score of 1–7. It is hypothesized that higher scores will be observed in the intervention group. Previous international studies have shown significant effects of parental sensitivity and structuring as well as child involvement [50], which also seem to be the subscales most indicative of maternal anxiety in the NICU [66].

The *secondary outcome measurements* are listed below. For measuring the cognitive, language, and motor development of the children, the Bayley Scales of Infant and Toddler Development, Third Edition (BSID-III) [67], will be used at 24 months corrected age. Composite scores are standardized to mean (SD) scores of 100 [15], based on age-matched normative data. The secondary hypothesis is that the mean will be higher in the intervention group.

The child’s executive function at 24 and 36 months corrected age will be measured with the Behaviour Rating of Executive Function, Parental Version (BRIEF-P) [68, 69]. All 5 subscales (*inhibit, shift, emotional control, working memory and plan/organize)* will be used, and the hypothesis is that the intervention group will have fewer executive problems reported.

Child’s motor development will be measured by the Alberta Infant Motor Scale (AIMS) [70, 71] at 3 and 12 months corrected age. The range of the AIMS is 0–58 points, with a hypothesis of higher scores for the intervention group.

Parental depression will be measured by Hospital Anxiety and Depression Scale (HADS) [72, 73] at term age and at 12, 24 and 36 months corrected age. On the HADS, the range for the depression subscales is 0–21, and the range for the anxiety subscales is 0–21. It is hypothesized that lower scores will be observed for the parents of the children in the intervention group.

Parental anxiety will be measured by the State-Trait Anxiety Inventory (STAI) [74] at term age and at 12, 24 and 36 months corrected age. Both the state and trait scales have a maximum score of 80 points. It is hypothesized that lower scores will be observed for the parents of the children in the intervention group.

Parental self-efficacy will be measured by the Parental Self-Efficacy Scale (PSE) [75] at term age and at 12, 24 and 36 months corrected age. The PSE has 24 items for children at term age and at 12 and 24 months corrected age, while it has 48 items for children of older ages. All items are rated on a 0–10 scale. It is hypothesized that higher scores will be observed for the intervention group at 12, 24 and 36 months corrected age.

Parental resilience will be measured by the Resilience Scale (RES) [76, 77] at term age and at 12, 24 and 36 months corrected age. The RES is a 25-item scale with a 7-point Likert scale. It is hypothesized that higher scores will be observed in the intervention group.

The *other outcome measurements* are listed below. The Hammersmith Neonatal Neurological Outcome (HNNE) is used at term age as a baseline measurement. Post discharge neurological development will be assessed with the Hammersmith Infant Neurological Examination (HINE) [78–80] at 3 months, 12 months, and 24 months corrected age. It is hypothesized that higher scores will be observed for the intervention group. The HINE can be used for infants 2–24 months of age and has optimal scores as well as cut-off values for future less fortunate motor outcomes. It includes 26 items assessing posture, movements, muscle tone, cranial nerve reflexes and reactions, with a score range of 0–3 for each time, thus yielding a possible sum of 0–78 points.

Children’s motor development will be measured using Peabody Developmental Motor Scales (PDMS) [81] at 12 months corrected age. It is hypothesized that higher scores will be observed for the intervention group. The ranges for each subscale are as follows: stationary, 0–42; locomotion, 0–138; object manipulation, 0–30; grasping, 0–44; and visual-motor integration, 0–113. It is hypothesized that higher scores will be observed for the intervention group.

Children’s neurological development will be measured using the General Movement Assessment (GMA) scale (normal-absent fidgety) at 3 months corrected age [82–84].

Children’s general development will be measured using the Ages and Stages Questionnaire (ASQ-R) [85–87] at 12, 24, and 36 months corrected age. It is hypothesized that the parents of the children in the intervention group will score their children higher. All five subscales (communication, gross motor, fine motor, problem solving, personal-social) will be used, with an overall score ranging from 0 to 300. It is hypothesized that higher scores will be observed for the intervention group.

Children’s strengths and difficulties will be measured using the Strengths and Difficulties Questionnaire (SDQ) [88, 89] at 24 and 36 months corrected age, with the hypothesis that fewer difficulties and more strengths will be scored by parents in the intervention group. The SDQ consists of 25 items on a 3-point scale: 5 items on prosocial behaviour and 20 questions about various difficulties. It is hypothesized that higher scores will be observed for the intervention group for prosocial behaviour and that lower scores will be observed for the intervention group on the problematic subscales.

Child’s autistic symptoms will be measured using the Modified Checklist for Autism in Toddlers (M-CHAT) [90] at 24 months corrected age. The scale ranges from 0 to 20 points, and it is hypothesized that lower scores will be observed for the intervention group.

Infant temperament is measured using the Infant Behaviour Questionnaire (IBQ-R) [91, 92] at 12 months corrected age. The IBQ-R consists of 37 items on a 7-point scale, and it is hypothesized that less problematic behaviour will be observed for the intervention group, i.e., the intervention group will have higher scores on smiling, laughter and soothability subscales and lower scores on the fear and distress to limitations subscales.

Parental satisfaction with the intervention is measured using the Client Satisfaction Questionnaire (CSQ-8) [93] and a semi-structured interview at 12 months corrected age. The CSQ-8 has 8 items and a range of 8–48 points.

Preschool educators’ views of the children’s engagement in preschool is measured using the Child Engagement Questionnaire (CEQ) [94, 95] at 24 and 36 months corrected age. The Swedish version of the CEQ has 29 items rated on a 4-point scale, and the summary score may range from 29 to 116, with higher scores indicating more positive engagement.

Preschool educators’ views of the children’s interaction in preschool will be measured using the Swedish questionnaire Ert Barn Vårt Samspel (EBVS) [94] at 24 and 36 months corrected age. The questionnaire has 36 items rated on a 5-point scale, and the summary score may range from 36 to 180, with higher scores indicating more interactive behaviour.

Preschool educators’ views of the children’s playtime in preschool will be measured using the play time/social time teacher impression scale [96, 97] at 24 and 36 months corrected age. The teacher impression scale has 16 items rated on a 1–5 Likert scale (overall score range: 16–80), with higher scores indicating more social skills and play behaviour. It is hypothesized that higher scores will be observed for the intervention group.

Preschool educators’ views of the children in preschool will be captured using a semi-structured preschool teacher interview at 24 or 36 months corrected age, depending on when the child has entered preschool.

Preschool educators’ views of the children’s level of functioning in preschool were captured with the ICF-CY core sets [94] at 24 and 36 months corrected age. The ICF-CY has 12 items on body functions (rated 0–9) and 22 items (rated 0–9) on activities and participation; higher scores indicate disability or developmental delay. Twenty items covering environmental factors (between + 4 and + 1 for facilitators; 0–9 for barriers) were included to identify possible disability and environmental moderators.

### Statistical analysis plan

Data will be analysed using intention to treat analysis for the primary outcome and separate testing with multiplicity adjustments for secondary outcomes. Data will be analysed using SPSS version 25 (IBM, New York) and reported according to the CONSORT statement for RCTs. Descriptive parametric statistics will be presented as percentage for categorical variables and as mean and SD for continuous variables such as age or centiles if the data is skewed. Comparisons between the two groups will be performed with Mann-Whitney U test for independent samples. Subgroups with additional analyses will also be used for detaching outcome differences according to number of home-visits in total, additional medical diagnoses and whether one or two parents participated in the intervention. The level of significance is specified at 0.05. To account for repeated measures, to model within-subject variance, and to handle correlated data of continuous variables a linear mixed model will be used. An interaction term will be introduced in the model to examine heterogeneity effect. For binary and ordinal outcome variables Generalized Estimating Equation (GEE) will be employed. For the main analysis no missing data will be imputed. However, classical multiple imputation methods will be used for an additional sensitivity analysis if any of the included variables has more than 5% missing observations. The GEE is a technique which produces unbiased estimates under the assumption that missing observations will be missing at random. An amended approach of weighted GEE will be employed if missingness is found not to be at random. We will perform residual analysis to assess model assumptions and goodness-of-fit.

Generalized linear modelling will be used for outcome variables that are not repeated to create regression models with distribution of the binary and ordinal dependent variables.

The Bonferroni method will be used to appropriately adjust the overall level of significance for multiple comparisons. Count variables will be analyzed using the Poisson regression model. Qualitative variables from the semi-structured interviews will be analysed for their thematic content. The Pearson chi-square test will be used to detect associations between categorical variables, and the mean and standard deviation will be presented for normally distributed continuous variables.

### Ethics and dissemination

The study was approved by the Regional Ethical Review Board in Stockholm (ref. 2017/1596–31). Since the SPIBI is an RCT, half of the participants will be randomized to the control condition, which entails an extended follow-up programme, as well as inquiries about parental mental health and resilience. To some, such questions may feel intrusive; on the other hand, answering them truthfully may open a possible channel of support and, if needed, a referral to professional help. The same clinically oriented approach will apply to developmental assessments of the children and the parent-child interactions.

In case the intervention does not have any statistically significant effects, it may be argued that all the time spent on the intervention was useless and could have been much more wisely spent on the parent-child dyad. However, the fact that the intervention is strengths-based is an ethical advantage even in the absence of the hypothesized effect. The research group is aware that ethical questions may arise at any time during the project and are prepared to identify and resolve them.

If the intervention has positive effects, there may be an ethical dilemma concerning the children in the control group, who will not benefit from the intervention. Since the intervention is age-specific, a wait-list design is not applicable. However, the control group will receive an extended follow-up programme that is intended to give the participants an extra sense of care and an opportunity for further referrals if needed.

The results will be disseminated through academic journals and presentations at research conferences. Since the research group consists of professionals from different parts of the Stockholm healthcare system, the results will easily reach clinical practitioners of neonatal, physiotherapeutic and child psychiatric care in Stockholm.

## Discussion

The SPIBI is an ongoing randomized controlled study, with an anticipated date for the cessation of recruitment of 31 August 2020. The importance of transparent research processes to facilitate control and replication will be supported by this protocol. This protocol is also intended to be shared with different healthcare professionals throughout the EPT care chain, making a unified approach through specialties possible. If we can show that this post-discharge early intervention in the EPT group affects parent-child interactions, child development and/or parental mental health in a positive way, this kind of programme could be introduced at a national or even cross-national level.

The EPT infant is often referred to as the most vulnerable patient in the hospital due to these infants’ immature bodies in general and sensitive brains in particular [98]. Brain plasticity continues throughout life, but the brain of the newborn infant is in an unceasing process of development and is utterly sensitive to disturbances. The sensitivity of the newborn brain poses a great potential risk when the EPT infant must live through stressful and painful medical procedures at the beginning of life, but the plasticity of the very same brain may potentially make it possible for these infants to experience the large positive effects of early interventions in the long run. Several researchers have argued that *“[t]here is evidence that intervention in the earliest years of life provides the greatest social and economic benefits to the individual, their family and the wider community*” [99]. Hence, the first year at home is an optimal time for early intervention in the EPT population.

One strength of the intervention is that its focus is three-sided, as the SPIBI aims to affect parent-child interactions, the individual child and the parents. The importance of reducing parental depression as well as general parental stress to benefit the development of the child cannot be overstated [37, 38, 100, 101]. The severity of prematurity outcomes has been shown to affect maternal well-being during the first year [102] and later in life [103].

A further strength of the trial is its multidisciplinary foundation, both in terms of researchers and interventionists. Since the risks of extremely preterm birth affect several parts of the child’s future development, relating to, i.e., cognitive, motor, social, psychiatric and academic areas, a broad approach to intervention makes sense. At a national level and for several years, the Swedish government has published reports with clear demands for increased cooperation among different healthcare professions [104], but such initiatives are still rare. There are several medical, psychological and economic reasons for this international and national focus on multidisciplinary teamwork. Two of the main medical reasons are that the patient is a whole organism and is not separated into subsystems, and there is evidence that the psychological and social circumstances of a preterm child will affect his or her general long-term outcomes [11, 45, 105–107]. There are constant economic implications for today’s healthcare, and with a growing population and increased survival rates of EPT children, it is no longer sustainable to divide care efforts, which leave a growing number of families bewildered and insecure due to healthcare providers giving sometimes contradictory advice. A French review of cost-of-illness studies on prematurity concluded that the cost of extreme prematurity is 100,000 USD per child [108], which may suggest a need for cost-effective early interventions in this group.

Although Sweden has active neonatal care with early interventions initiated during the hospital stay and a world-renowned follow-up assessment programme [98, 109], no systematic post-discharge interventions have been implemented thus far. Until recently, the exclusive focus of neonatal care has been survival, but with increasing surviving levels as well as the national decision to save even more immature infants [16], the need to support development as well as parental mental health can no longer be overlooked. In conclusion, if the SPIBI shows positive effects on parent-child interaction, child development and/or parental mental health, there are child-, family- and society-based arguments for its implementation in clinical practice. However, even non-significant results can be of interest, since the first year at home for preterm children and their parents is an under-researched area in Sweden due to the previous focus on the NICU stay and discharge process.

## Data Availability

On an aggregated level, the SPIBI research-team may share aggregated data upon reasonable request. The manual for the intervention is free to use for any other research team after information and education from the SPIBI research team.

## Appendix

**Table 4:** WHO Trial Registration Data Set (Version 1.3.1). Stockholm Preterm Interaction-Based Intervention

Trial information	Statues of SPIBI		
Primary Registry and Trial Identifying Number	Clinical Trials.gov NCT03714633		
Date of Registration in Primary Registry	22nd of October 2018		
Secondary Identifying Numbers	No protocol number so far. Manual: TiSam – Tidigt Samspel för prematurfödda barn och deras föräldrar: Interventionunderlag		
Source (s) of Monetary or Material Support	Stockholm University, Sweden, department of Special Education, through faculty funds. Karolinska Institutet – department of Women’s and Children’s health, Sweden Stockholm County Council through the collaboration program with Stockholm University 2017–2019 (SU-SLL no. 20160881) Centrum för kompetensutveckling inom vård och omsorg at Stockholm University (CKVO) 2018–19 (no. SU FV 2.1.1–402,417) Clas Groschinskys Minnesfond 2018 (No. SF 18109) Queen Silvia Jubilee Fund for research on children and disability (date of letter of acceptance 13th of December 2017) Filénska fonden 2017/2018 K & A Wallenberg foundation (no. SU FV 2..1.9.1894–18) Lilla Barnets fond (2019-10-01)		
Primary Sponsor	Stockholm University		
Secondary Sponsor (s)	Karolinska Institutet		
Contact for Public Queries	Erika Baraldi, PhD student Stockholm University, erika.baraldi@specped.su.se + 46,812,076,462		
Contact for Scientific Queries	PI: Ulrika Ådén, professor of neonatology Karolinska Institutet ulrika.aden@ki.se + 46,852,480,000 Mara Westling Allodi, professor of Special Education Stockholm University mara.allodi@specped.su.se + 468,162,000, + 46,734,612,522		
Public Title	Tidigt samspelsbaserad intervention för extremt prematurfödda barn (TiSam)		
Scientific Title	Stockholm Preterm Interaction-Based Intervention (SPIBI); RCT of a 12-months parallel-group post-discharge program for extreme premature infants and their parents		
Countries of Recruitment	Sweden, Stockholm area		
Health Condition (s) or Problem (s) Studied	Extreme prematurity, parenthood of extremely preterm children		
Intervention (s)	Active comparator: Home-based post-discharge intervention for extremely premature infants and their parents. The intervention consists of one hospital visit, nine home-visits and two telephone calls during the first year corrected age, specifically from 1 week before discharge to 12 months corrected age. The intervention is strengths-based working with the infant-parent interaction, supporting infant development and strengthening the parent in his/her role.		
	Control condition: The participants of the Control Group receives treatment as usual, which consists of a regular follow-up program with neurodevelopmental assessment at term age, 3 months corrected age, 12 months corrected age, 24 months corrected age and 66 months corrected age. Compared to children not participating in the study, the control group will receive an extended follow-up program, with assessment and questionnaires at term age, 3 months corrected age, 12 months corrected age, 24 months corrected age and 36 months corrected age. Participants in the control group will be referred to specialized care when needed.		
Key Inclusion and Exclusion Criteria	Inclusion Criteria: • extremely premature born infants • close to discharge from their neonatal intensive care unit hospital stay at Stockholm county council (Stockholms Läns Landsting).		
	Exclusion Criteria: • Children with parent/parents who do not communicate in Swedish or English. • Patients not residing in Stockholm County. • Acute surgery patients who will spend a lot of time at hospitals far from Stockholm		
Study Type	Interventional		
	Study design Method; randomized Masking; Semi-masked, primary outcome assessor is blind to allocation Assignment; Parallel Purpose; The purpose of the SPIBI is to improve parent-child interaction, child development and parental mental health		
	Phase; Not fully applicable, Phase II/Phase III		
Date of First Enrollment	September 2018		
Sample Size	Planned: 130		
	Today (22nd of November 2019): 48		
Recruitment Status	Recruiting		
Primary Outcome (s)	Emotional Availability of parent and child measured with Emotional Availability Scales EAS.		
	Time point: 12 months corrected age		
	Method: Video Observation of 20 min parent-child interaction, assessed with the manualized method of EAS consisting of 4 parent-specific domains and 2 child-specific domains.		
Key Secondary Outcomes	Bayley scales of infant and toddler development third edition, BSID-III	24 months corrected age	Child assessment
	Behavior Rating of Executive Function Parental version BRIEF-P	24 and 36 months corrected age	Parent questionnaire of child behavior
	Alberta Infant Motor Scale, AIMS	3 and 12 months corrected age	Observation and assessment of child motor development
	Hospital anxiety and depression scale, HADS	12, 24 and 36 months corrected age	Parent questionnaire of depressive symptoms
	State/trait anxiety inventory, STAI	12, 24 and 36 months corrected age	Parent questionnaire of symptoms of anxiety
	Parental self-efficacy scale, PSE	12, 24 and 36 months corrected age	Parent questionnaire of parental self-efficacy
	Resilience scale, RES	12, 24 and 36 months corrected age	Parent questionnaire of parental self-efficacy
Ethics Review	SPIBI has been approved by the Regional Ethical Review Board in Stockholm ref. 2017/1596–31.		
	Date of Approval 26th of October 2019.		
	Contact: Göran Elinder, scientific secretary, kansli@stockholm.epn.se + 46,852,487,000		
Completion date	Last day of recruitment: 31st of August 2020 (anticipated), may be later if target is not reached yet.		
	Last visit: 31st of August 2021 (anticipated) or 1 year after the ast participant has been recruited.		
	Data collection completion: 1st of September 2023 (anticipated)		
Summary Results	No results yet		
IPD sharing statement	Undecided		

## Abbreviations

ADHD: Attention Deficit Hyperactivity Disorder
AIMS: Alberta Infant Motor Scale
ASD: Autism Spectrum Disorder
ASQ: Ages and Stages Questionnaire
BRIEF-P: Behavior Rating Inventory of Executive Function – Parental Version
BSID: Bayley Scales of Infant and Toddler Development
CA: Corrected Age
CEQ: Child Engagement Questionnaire
CSQ: Client Satisfaction Questionnaire
EAS: Emotional Availability Scale
EBVS: Ert Barn Vårt Samspel
EPT: Extremely Preterm
GEE: Generalized Estimated Equation
GMA: General Movement Assessment
GW: Gestational Weeks
HADS: Hospital Anxiety and Depression Scale
HINE: Hammersmith Infant Neurological Examination
HNNE: Hammersmith Neonatal Neurological Examination
IBAIP: Infant Behavioral Assessment and Intervention Program
IBQ: Infant Behavior Questionnaire
ICF-CY: International Classification of Functioning, Disability and Health for Children and Youth
IHDP: Infant Health and Development Program
ITT: Intention To Treat
M-CHAT: Modifierad Checklista för Autism hos små barn (Autism Checklist for Small Children)
MITP: Mother Infant Transaction Program
NICU: Neonatal Intensive Care Unit
NIDCAP: Newborn Individualized Developmental Care and Assessment Program
PDMS: Peabody Developmental Motor Scales
PSE: Parental Self-Efficacy
RCT: Randomized Controlled Trial
RES: Resilience Scale
SDQ: Strengths and Difficulties Questionnaire
SPF: Svenska Prematurförbundet (Swedish Federation for Preterm Infants)
SPIBI: Stockholm Preterm Interaction-Based Intervention
STAI: State-Trait Anxiety Inventory
